# Live birth rates following individualized dosing algorithm of follitropin delta in a long GnRH agonist protocol

**DOI:** 10.1186/s12958-023-01090-w

**Published:** 2023-05-16

**Authors:** Manuel Fernández Sánchez, Per Larsson, Marcos Ferrando Serrano, Ernesto Bosch, Juan Antonio García Velasco, Esther Santamaría López, Bernadette Mannaerts

**Affiliations:** 1 IVI-RMA Seville, Avenida Americo Vespucio 19, Seville, ES-41092 Spain; 2 Department of Surgery, Universidad de Sevilla, Seville, ES-41004 Spain; 3 Department of Molecular Biology and Biochemical Engineering, Universidad Pablo de Olavide, Seville, 41013 ES Spain; 4 Fundacion IVI, Instituto Investigación Sanitaria La Fe, Valencia, ES-46026 Spain; 5 Global Biometrics, Ferring Pharmaceuticals A/S, Amager Strandvej 405, Kastrup, 2770 Denmark; 6 IVI Bilbao, Bilbao, Valencia Spain; 7 IVI Valencia, Valencia, Spain; 8 IVI-RMA Madrid, Madrid, Spain; 9 Reproductive Medicine & Maternal Health, Ferring Pharmaceuticals A/S, Amager Strandvej 405, Kastrup, 2770 Denmark

**Keywords:** Long GnRH agonist, Individualized dosing, Follitropin delta, Cumulative live birth rates

## Abstract

**Purpose:**

To explore the efficacy and safety of individualized follitropin delta dosing, based on serum anti-Müllerian hormone (AMH) concentration and bodyweight, in a long gonadotropin-releasing hormone (GnRH) agonist protocol.

**Methods:**

Clinical outcomes after one treatment cycle are reported in women with AMH: 5–35 pmol/L. Oocytes were inseminated by intracytoplasmic sperm injection, blastocyst transfer was on Day 5 and remaining blastocysts were cryopreserved. Data collection included live births and neonatal health follow-up for all fresh/frozen transfers performed within one year after treatment allocation.

**Results:**

In total, 104 women started stimulation, of whom 101 had oocyte recovery and 92 had blastocyst transfer. The average daily dose of follitropin delta was 11.0 ± 1.6 µg and the duration of stimulation was 10.3 ± 1.6 days. The mean number of oocytes was 12.5 ± 6.4, the mean number of blastocysts was 5.1 ± 3.4, and 85% had at least one good-quality blastocyst. Following mostly single blastocyst transfer (95%), the ongoing pregnancy rate was 43%, the live-birth rate was 43%, and the cumulative live-birth rate was 58% per started stimulation. There were 6 cases of early OHSS (5.8%) graded as mild (n = 3) and moderate (n = 3) and 6 cases of late OHSS (5.8%) graded as moderate (n = 3) and severe (n = 3).

**Conclusion:**

In this first evaluation of the individualized follitropin delta dosing in a long GnRH agonist protocol, the cumulative live-birth rate was high. A randomized trial comparing follitropin delta in a long GnRH agonist protocol versus in a GnRH antagonist protocol should provide further insight into the efficacy and safety of this treatment option.

**Trial registration number:**

NCT03564509; June 21, 2018.

## Introduction

Gonadotropin Releasing Hormone (GnRH) agonists have been used for many years and are still applied for the prevention of premature luteinizing hormone (LH) surges in patients undergoing ovarian stimulation (OS) prior to Assisted Reproductive Technologies (ART). Before the development of GnRH antagonists for this indication, a long GnRH agonist protocol with conventional follicle stimulating hormone (FSH) dosing was the standard therapy for in vitro fertilization (IVF) patients undergoing OS. Such protocol requires at least 10 days of treatment to obtain pituitary desensitization and FSH stimulation may induce a higher ovarian response and a higher risk of ovarian hyperstimulation syndrome (OHSS) than with GnRH antagonist protocols [1]. Since the development of the GnRH antagonists for the prevention of premature LH surges, most patients are treated with a GnRH antagonist protocol which is short, simple and convenient for the patient [2]. Randomized clinical trials have demonstrated that the GnRH antagonist protocol recruits a smaller number of follicles with lower serum oestradiol and, therefore, the risk of OHSS following triggering of final follicular maturation with human choriogonadotropin (hCG) is lower [3]. Moreover, in high responders the risk of OHSS can be largely mitigated by triggering final follicular maturation with a GnRH agonist instead of hCG [4].

OS protocols have improved over recent years by stepping away from conventional dosing and focusing on the individual needs of each patient. Follitropin delta, a novel recombinant FSH (rFSH) preparation, has been developed with an individualized dosing regimen based on each woman’s serum anti-Müllerian hormone (AMH) concentration and bodyweight. This dosing regimen is to guide clinicians to achieve an ovarian response that can minimize the risk of OHSS without compromising efficacy. The dosing algorithm was built upon the pharmacokinetic/ pharmacodynamic data of a Phase 2 dose-response trial in IVF patients using a GnRH antagonist protocol [5, 6]. Subsequently, the dosing algorithm was prospectively tested and validated in several comparative trials using a GnRH antagonist protocol [7–9]. These studies demonstrated the efficacy of individualized follitropin delta dosing compared with conventional rFSH treatment with respect to ongoing implantation/pregnancy rate and live births. Individualized follitropin delta dosing was additionally shown to provide a reduction in iatrogenic complications and a reduction in the need for preventive interventions of OHSS [10]. In the most recent completed Pan-Asia trial, OS with individualized follitropin delta dosing resulted in significantly higher live birth rate and a significantly lower incidence of early ovarian hyperstimulation syndrome (OHSS) and/or preventive interventions compared with conventional follitropin alfa dosing [9]. This improved clinical outcome pertained most to the younger IVF patients included in this Pan-Asia trial who benefitted most from this individualized dosing regimen, as their AMH was relatively high and their bodyweight relatively low.

Following the development of the individualized follitropin delta dosing in a GnRH antagonist protocol, it was questioned whether this specific dosing algorithm could also be applied in a long GnRH agonist protocol. The long GnRH agonist protocol is still applied frequently for selected indications such as low responders. In some large IVF clinics, the long GnRH agonist may be preferred to manage patients in cohorts during stimulation and to avoid oocyte recoveries during the weekend [11]. The most recent meta-analysis showed that long GnRH agonist protocols did not result in a higher live birth rate than the GnRH antagonist protocols among general IVF populations, but came with a higher risk of OHSS, which is in agreement with the higher ovarian response following a long agonist protocol [1, 12]. The first application of follitropin delta in a long GnRH agonist protocol was in a prospective Phase 2 dose-range trial of choriogonadotropin (CG) beta in which potential high responders were excluded [13]. Here, we report on the cumulative live birth rate of 104 patients in the control (placebo) group from the Phase 2 trial who underwent one treatment cycle with individualized follitropin delta (without CG beta) in a long GnRH agonist protocol.

## Materials and methods

### Study design and participants

This is a subset analysis including all 104 patients participating in a European Phase 2 dose-range trial and treated with individualized follitropin delta dosing plus placebo in a long GnRH agonist protocol as previously described [13]. The trial was approved by the local regulatory authorities and the independent ethics committees covering all participating centres, and was performed in accordance with the principles of the Declaration of Helsinki, the International Conference on Harmonization Guidelines for Good Clinical Practice, and local regulatory requirements.

Women (30–42 years) who were undergoing their first or second IVF/intracytoplasmic sperm injection (ICSI) cycle due to unexplained infertility, tubal infertility, endometriosis Stage I/II, or with partners diagnosed with male factor infertility, were eligible for the trial. Additional main inclusion criteria were body mass index 17.5–32.0 kg/m^2^, regular menstrual cycles of 24–35 days and AMH levels at screening of 5.0–35.0 pmol/L as measured by Elecsys^®^ AMH Plus Immunoassay (Roche, Switzerland) at a central laboratory.

### Ovarian stimulation in a long GnRH agonist protocol

Women started treatment with subcutaneous triptorelin acetate 0.1 mg/day (Gonapeptyl^®^, Ferring Pharmaceuticals, Switzerland) during the mid-luteal phase of their menstrual cycle. In total, 104 pituitary down-regulated women were allocated to the placebo group (i.e., were given placebo instead of choriogonadotropin beta) and received an individualized fixed daily dose of follitropin delta, determined on the basis of their AMH level at screening and their bodyweight at stimulation Day 1. During stimulation, subjects were monitored by transvaginal ultrasound on stimulation Days 1 and 6 and thereafter at least every second day. Triggering of final follicular maturation was done as soon as ≥ 3 follicles with a diameter of ≥ 17 mm were observed. If there were < 25 follicles with a diameter ≥ 12 mm, a single dose of rhCG 250 µg (Ovitrelle^®^, Merck Europe, Netherlands) was administered, but if there were ≥ 25 follicles with a diameter ≥ 12 mm, the cycle had to be cancelled per protocol. Oocyte retrieval took place 36 ± 2 h after triggering, the maturity of each oocyte was assessed and each metaphase II oocyte was inseminated by ICSI. All inseminated oocytes were incubated in an EmbryoScope® (Vitrolife, Sweden) for time-lapse monitoring and embryo development was recorded up to the day of transfer.

Assessment of blastocyst quality on Day 5 after oocyte retrieval was done locally and centrally and consisted of assessment of three parameters: blastocyst expansion and hatching status (Grade 1–6), blastocyst inner cell mass grading (Grade A–D), and trophectoderm grading (Grade A–D) [14]. Blastocysts were scored by using the system of Gardner D and Schoolcraft W [15], with the addition of Category D  for scoring inner cell mass and trophectoderm. For all subjects, fresh blastocyst transfer was performed on Day 5 after oocyte retrieval. Single blastocyst transfer was mandatory for subjects ≤ 37 years. In subjects ≥ 38 years, the transfer policy was dependent on the quality of the available blastocysts, i.e., single blastocyst transfer if they had at least one good-quality blastocyst, and transfer of maximum 2 blastocysts if they had no good quality blastocysts. Remaining blastocysts were cryopreserved and transferred within 1 year after treatment allocation.

Pre-implantation genetic diagnosis/pre-implantation genetic screening was prohibited by the trial protocol. Vaginal progesterone tablets 100 mg (Lutinus^®^, Ferring, Switzerland) three times daily were provided for luteal phase support from the day after oocyte retrieval until the ongoing pregnancy visit. A serum βhCG test was performed 13–15 days after transfer, clinical pregnancy was confirmed by transvaginal ultrasound 5–6 weeks after transfer, and ongoing pregnancy was confirmed by transvaginal or abdominal ultrasound 10–11 weeks after transfer. The safety endpoints included OHSS, which was reported based on categorization of mild, moderate or severe OHSS by Golan’s classification system [16].

### Follow-up of cryopreserved embryos and neonatal health

Women initiating cryopreserved cycles were part of the follow-up trial. This interval was extended for approximately 3 months to compensate for a temporary deferral of cryopreserved cycles during the COVID-19 pandemic. A maximum of 2 blastocysts could be transferred in a cryopreserved cycle. Data gathered included positive βhCG test, clinical pregnancy, vital pregnancy, ongoing pregnancy as well as live birth rate. All pregnant women were followed until delivery to gather data on neonatal health, including minor/major congenital anomalies, at birth and 4 weeks after birth. Congenital malformation was adjudicated into major or minor in accordance with the EMA guideline (2005) [17].

### Statistical analysis

Pregnancy and live birth data were described using summary statistics and graphical illustrations. The cumulative live birth rate was calculated as the percentage of women starting stimulation who had one live born neonate. Pregnancy and live birth rates per cryopreserved transfer were calculated based on all the cryotransfers that were performed and therefore the same women could contribute data from more than one transfer. Rates per transfer were also estimated using a mixed-effects model, adjusting for repeated transfers within the same women, but since the variance component in this model was estimated close to zero the adjusted estimates were identical to the observed rates.

## Results

### Disposition and baseline characteristics

In total, 104 women were downregulated in a long GnRH agonist protocol of daily triptorelin 0.1 mg and started stimulation with individualized follitropin delta dosing. Their mean age and bodyweight were 35.6 ± 3.2 years and 64.1 ± 9.1 kg, respectively (Table 1). In total, 57.7% of women had an AMH < 15 pmol/l and 42.3% of women had an AMH ≥ 15 pmol/L. The proportion of women with primary infertility was 56.7% and the mean duration of infertility was 33.2 ± 21.9 months. The most common primary reasons for infertility were unexplained infertility (58.7%) and infertility related to male factor (28.9%).

**Table 1:** Demographics and baseline characteristics

Patient characteristic	N = 104
**Age (years)**, mean ± SD	35.6 ± 3.2
**Bodyweight (kg)**, mean ± SD	64.1 ± 9.1
**Body mass index (kg/m**^**2**^**)**, mean ± SD	23.6 ± 3.1
**Body mass index category**, n (%)	
<18.5 kg/m^2^	3 (2.9)
≥18.5 kg/m^2^ and < 25.0 kg/m^2^	73 (70.2)
≥25.0 kg/m^2^ and < 30.0 kg/m^2^	23 (22.1)
≥30.0 kg/m^2^	5 (4.8)
**AMH (pmol/L)**, mean ± SD	14.8 ± 6.9
**Primary infertility**, n (%)	59 (56.7)
**Duration of infertility (months)**, mean ± SD	33.2 ± 21.9
**Primary reason(s) for infertility**, n (%)	
Unexplained infertility	61 (58.7)
Tubal infertility	11 (10.6)
Male factor	30 (28.9)
Other	1 (1.0)
Missing	1 (1.0)

All women who started daily triptorelin treatment were successfully downregulated after 2 weeks without cyst formation. The total duration of GnRH agonist treatment was 25.5 ± 2.8 days. Of the 104 women who started stimulation, 3 women had oocyte collection cancelled (two women with too low ovarian response and one woman with a skin rash). In total, 101 women underwent oocyte retrieval (97%), 92 women (88%) had fresh blastocyst transfer which included 87 single transfers and 5 double transfers. Twenty-two women had one cryotransfer, ten women had two, four women had three, and one woman had four cryotransfers. Therefore, in total thirty-seven women had between 1 and 4 cryotransfers and the total number of cryotransfers was 58 (56 single transfers and 2 double transfers).

### Ovarian stimulation outcomes

The mean ± standard deviation duration of stimulation was 10.3 ± 1.6 days, the assigned follitropin delta dose was 11.0 ± 1.6 µg (range: 6.33–12 µg) and 64% received the maximum approved dose of 12 µg. At the end of stimulation, the mean number of follicles ≥ 12 mm was 12.7 ± 5.3 and the mean number of follicles ≥ 17 mm was 5.2 ± 2.4 with mean levels of serum oestradiol and progesterone of 5959 pmol/L and 1.34 nmol/L, respectively. The mean total number of oocytes and MII oocytes was 12.5 ± 6.4 and 9.7 ± 5.2, respectively, whereas the fertilization rate was 76.2%. The mean number of blastocysts, good-quality blastocyst and cryopreserved blastocysts was 5.1 ± 3.4, 3.7 ± 3.0 and 3.3 ± 3.0, respectively, and 85% of women who started stimulation had at least one good quality blastocyst at Day 5 or 6.

### Pregnancy outcomes

In total, there were 45 ongoing pregnancies following fresh blastocyst transfer. After fresh transfer, the positive βhCG rate was 50.0%, the vital pregnancy rate was 43.3%, and the ongoing pregnancy rate was 43.3% per started cycle and 48.9% per transfer. In addition, 15 ongoing pregnancies were established in cryopreserved cycles and the ongoing pregnancy rate per cryopreserved transfer was 25.9% resulting in a final cumulative ongoing pregnancy rate of 57.7%. There were 16 early pregnancy losses between the positive βhCG test and ongoing pregnancy for fresh (eight losses) and cryopreserved (eight losses) transfers.

### Live births

**Fig. 1:**
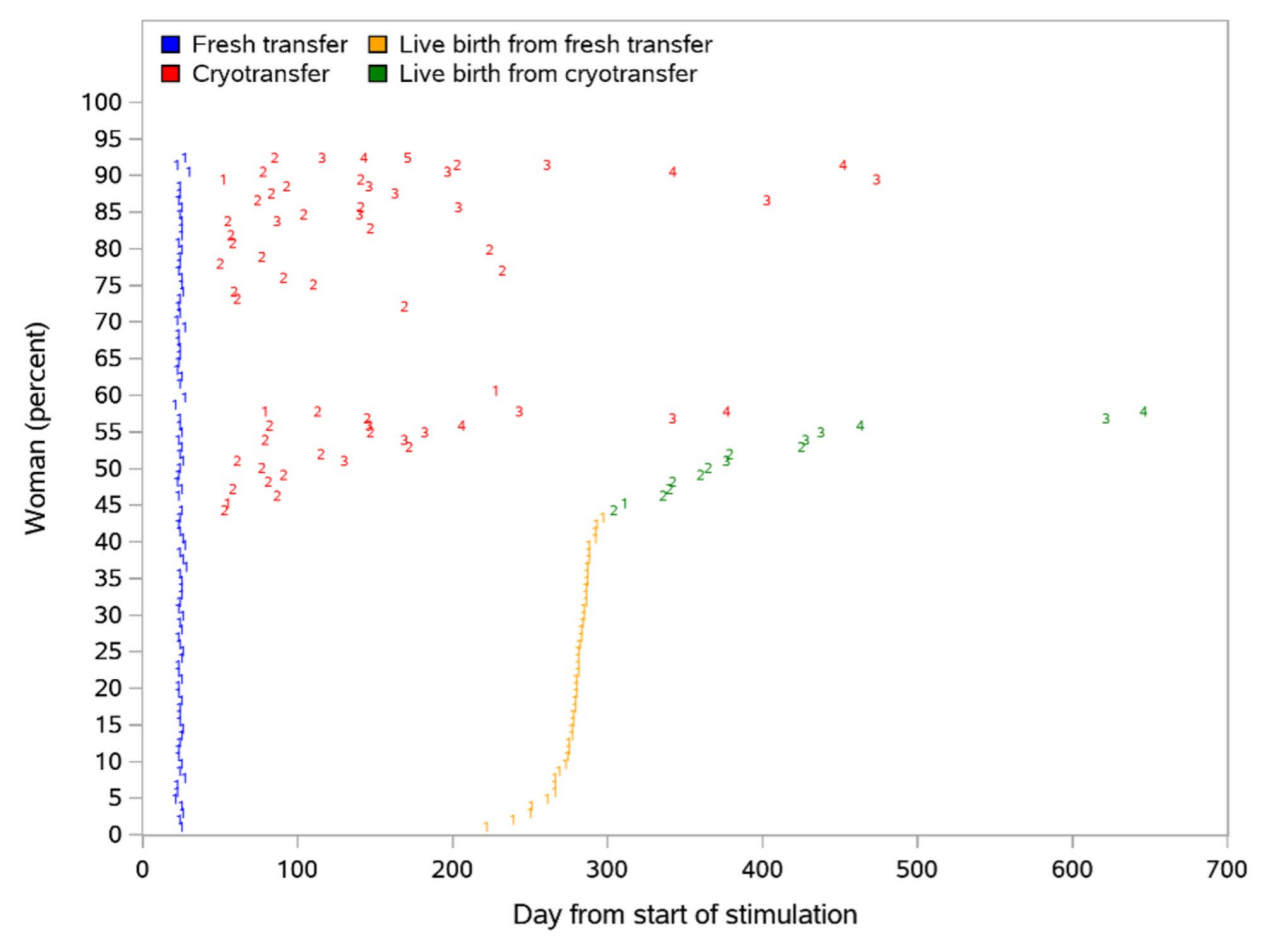
All fresh and cryopreserved transfers, and live births, versus time from start of stimulation. Digits represent the order of the transfers, with 1 being the first transfer (fresh, or the first cryopreserved for subjects without fresh transfer), and 2 being the second transfer (cryopreserved), etc. The subjects are sorted by time to first live birth, and shown as percent of all subjects, so that the live birth digits form a cumulative live birth rate vs. time curve

**Fig. 2:**
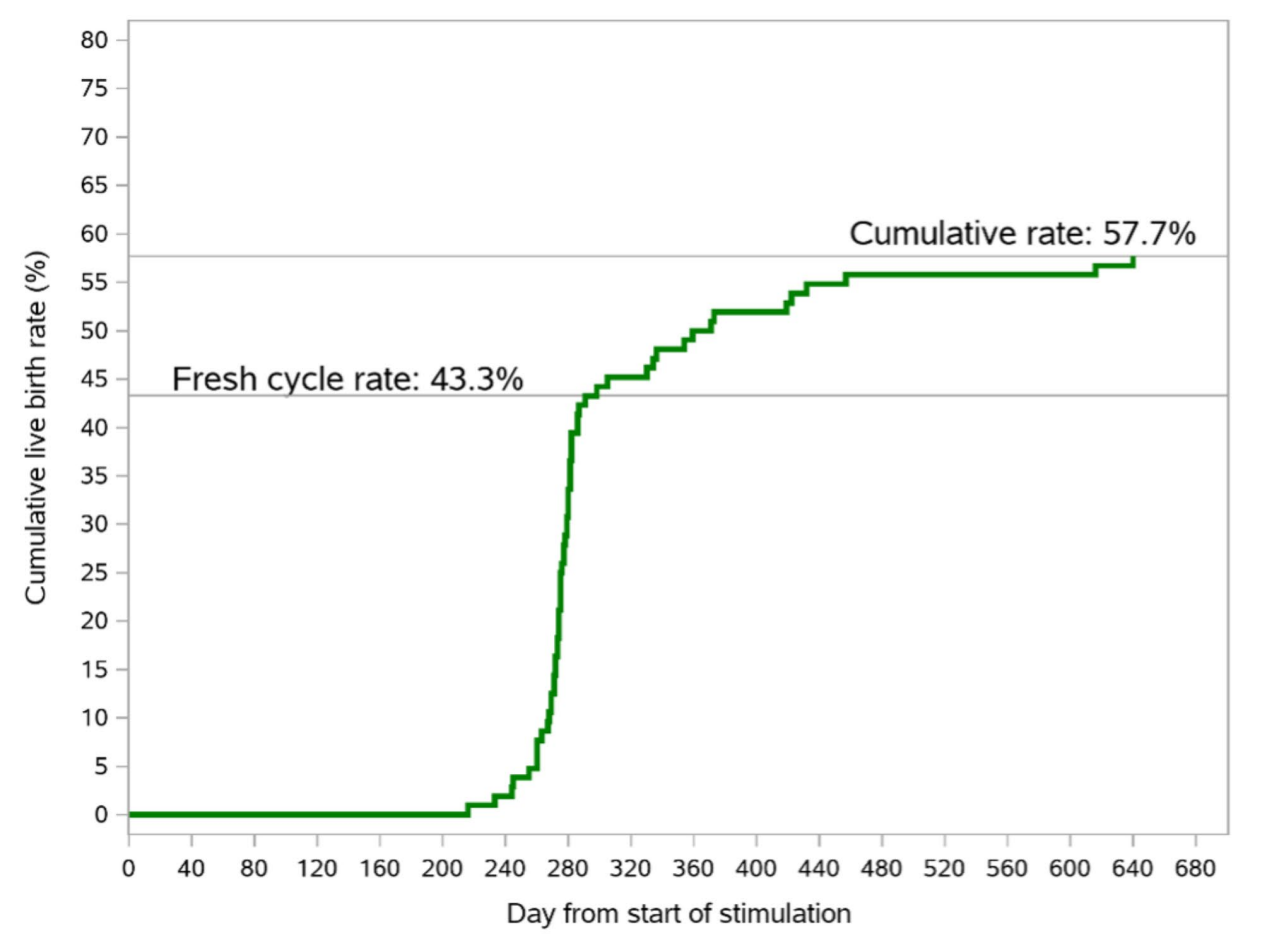
Cumulative live birth rate versus time from start of stimulation. Following fresh blastocyst transfer, 43.3% of women had live birth and following cryopreserved transfers the live birth rate further increased to 57.7% (both at birth and 4 weeks after birth)

None of the established ongoing pregnancies were lost prior to birth and all pregnancies resulted in live-born singletons. Figure 1 illustrates the timepoint (days after randomization) for all fresh and cryopreserved transfers and all live births from fresh and cryopreserved transfers. Figure 2 shows the cumulative live birth rate versus time, starting at the first day of stimulation. Following fresh blastocyst transfer, 43.3% of women had live birth and following cryopreserved transfers the live birth rate further increased to 57.7%.

### OHSS

There were in total 12 women (11.5%) with OHSS comprising 6 women with early onset OHSS and 6 pregnant women with late onset OHSS. Women with early onset had either mild OHSS (n = 3) or moderate OHSS (n = 3) whereas women with late onset OHSS had moderate OHSS (n = 3) or severe OHSS (n = 3), the latter including 2 hospitalizations. The 6 women with early onset OHSS were treated with doses of 10.33–12 µg/daily and their AMH ranged between 15 and 20 pmol/L (2 of these women received follitropin delta 12 µg/day and 4 received lower daily doses). Three women with mild OHSS did not develop ascites and 3 women with moderate OHSS showed ascites on ultrasound (3.5–19.2 cm^2^) without related clinical symptoms. The three women with early onset mild OHSS underwent fresh blastocyst transfer each resulting in a live birth.

### Infant follow-up

At birth there were 6 congenital malformations noted in 3 out of 60 singletons. All infants with malformations were from fresh blastocyst transfers. These included one major malformation (feet deformation) and 5 minor malformations (haemangioma, congenital cerebral cyst, hypospadias, atrial septal defect and patent ductus arteriosus). No additional congenital malformations were detected during the first 4 weeks after birth.

## Discussion

This is the first report on the cumulative live birth rate following one stimulation cycle with individualized follitropin delta in a long GnRH agonist protocol. This success rate (57.7%) is high considering that women with AMH > 35 nmol/L were excluded from the trial and that the overall cumulative live birth rate after up to three stimulation cycles with individualized follitropin delta in a GnRH antagonist protocol was 60.3% [18]. Overall, the pregnancy and neonatal outcomes from fresh and frozen cycles in this cohort of 104 women supports the efficacy of individualized follitropin delta dosing algorithm when used in a long GnRH agonist protocol.

The individualized dosing regimen of follitropin delta, which is based on a patient’s AMH and bodyweight, has been developed in a GnRH antagonist protocol and applies a fixed daily dose during stimulation without dose adjustments. The follitropin delta dose is calculated for each individual patient and overall, less FSH is required to reach the same triggering criteria compared with conventional FSH dosing. Considering the FSH threshold/window concept of follicle development [19], it was uncertain whether individualized, fixed dosing of follitropin delta dosing would sustain multiple follicular development in all down-regulated patients equally well, but actually only 3 out of 104 patients who started stimulation were cancelled prior to oocyte recovery.

When it comes to OS prior to IVF or ICSI, intra-cycle dose adjustments have been reported in up to 45% of ART cycle [20]. Research with individualized, fixed dosing of follitropin delta in comparison to conventional dosing including dose adjustments during stimulation of follitropin alfa has proven that the starting dose is most important [7]. Thus, once the optimal follitropin delta dose for a specific patient is selected, there is no need to adjust the dose during stimulation which simplifies treatment both in GnRH antagonist and long GnRH agonist cycles. This is in good agreement with previous findings that increasing the dose during stimulation does not lead to more oocytes [21, 22] making the starting dose critical for the ultimate ovarian response during that cycle.

In the current study, the average number of oocytes (12.5) and the number of good-quality blastocysts (3.7) tended to be higher than in previous follitropin delta trials using a GnRH antagonist protocol in which women with AMH > 35 pmol/L were not excluded [7–9]. In fresh cycles, most of the transferred blastocysts were of Grade 5AA or 5AB, whereas in cryopreserved cycles most of the transferred blastocysts were of Grade 5BB and 4BB. This can partly explain the higher pregnancy rates for fresh transfers compared with cryotransfers. Another explanation is that cryotransfers were mainly performed in women that already had a fresh transfer without pregnancy, and therefore these women had a lower probability of achieving pregnancy. There was a clear association between the blastocyst quality score and the implantation rate, supporting that the recovery of high-quality blastocysts in this study contributed to the high cumulative pregnancy rate [23, 24].

One of the main disadvantages of a long GnRH agonist protocol is the higher risk of OHSS [1, 12] as well as the impossibility of triggering final follicular maturation with a GnRH agonist instead of administration of hCG [4]. Regardless of these well-documented findings, a long GnRH agonist protocol is still applied in certain European countries, and in China where the GnRH antagonist was introduced much later [25]. In this cohort of 104 women, there were in total 6 cases of mild to moderate OHSS with early onset and 6 pregnant women with late OHSS, the latter including 2 hospitalizations. This OHSS incidence appears higher than in a previous GnRH antagonist trial of individualized follitropin delta using the same OHSS classification [7]; however, any reliable comparison should be based on a randomized trial comparing the incidence of OHSS in a long GnRH agonist with that in a GnRH antagonist protocol and such trial has been recently completed (NCT03809429). Obviously, the risk of OHSS is directly related to the ovarian response [26, 27], but may be mitigated in a long GnRH agonist protocol by excluding potential high responders (e.g. those with AMH > 35 pmol/L) who benefit more from individualized follitropin delta treatment in a GnRH antagonist protocol [28]. In contrast, one may hypothesize that low to normal responders (e.g. those with AMH ≤ 15 pmol/L) treated with individualized follitropin delta in a long GnRH agonist protocol may have a higher ovarian response than in a GnRH antagonist protocol, whereas the risk of OHSS may not be increased due to the individualized FSH dosing.

In conclusion, this first report on individualized, fixed, follitropin delta dosing in a long GnRH protocol showed adequate OS without the risk of cancellation due to too high ovarian response which supports the correctly chosen upper AMH limit of 35 pmol/L in this trial. As in the GnRH antagonist protocol, individualized dosing of follitropin delta brings a new treatment option with potential advantages over conventional dosing in a long GnRH agonist protocol, but those need to be documented in further clinical research.

## Data Availability

The data underlying this article will be shared on reasonable request to the corresponding author.
